# The Treatment of Nervous Diseases in Syphilitic Patients

**Published:** 1873-09

**Authors:** 


### The Treatment of Nervous Disorders in Syphilitic Patients.

In concluding a series of articles on Paralysis, Convulsions and other Nervous Affections in Syphilitic subjects, in the *Lancet*, Dr. THOMAS BUZZARD, says:

" So much has been incidentally said in these papers respecting the treatment of diseases of the nervous system dependent upon syphilis that but little remains to be added upon this point. Ricord's views as to the most appropriate treatment for the different stages of constitutional syphilis may be thus epitomised. In the primary stage, mercury. For secondary sympt ms, when these are uncomplicated, mercury; when they are blended with those of a tertiary character, the treatment is a mixture of mercury and iodide of potassium. In the tertiary stage he relies upon iodide of potassium. And this scheme would probably represent very fairly the views of those who have had large experience of the disease. In the class of cases, however, with which we are specially concerned, as I have before described, it is not by any means always possible to say whether the symptoms of nervous affection which are observed belong to the secondary or tertiary stage. Our treatment therefore must be to a certain extent tentative. My own plan is always to commence, in a case of this kind, with iodide of potassium. In a majority of instances this drug, which will often require to be pushed to large doses, will be sufficient to bring about a cure of the symptoms, or at least a very important improvement. If, however, after three or four weeks of such treatment, employed with sufficient boldness, the improvement is not marked, I give mercury, which will sometimes act with remarkable effect. The preparations which I chiefly employ are, the perchloride and the red iodide; and I do not know that preference, for any particular reason, is to be given to one or other of these forms. As a matter of practice, I think I have found that the red iodide, given in solution of iodide of potassium, is less apt to cause irritation of the abdominal organs than the perchloride. It is very seldom that either causes salivation. Where it is desired to produce, and to keep up for some time a slight action on the gums, this can be effected in the usual manner by appropriate doses of blue pill or grey powder. The French very largely employ the green iodide, a preparation but seldom used in England, probably because it is unstable, some biniodide being apt to form in it when it has been long kept.

A word as to the dose of iodide of potassium. After a good deal of hesitation, and trial of various quantities in a considerable number of cases, I feel convinced that in syphilitic affections of the nervous system it is often necessary to employ doses of this drug which are far beyond those usually ordered. In several instances I have observed something of the following kind to take place. An improvement up to a certain point has been produced by doses of iodide of from ten or fifteen to twenty grains three times daily. The patient has then remained at this stage, or progress has been very slow, whilst continuing to take this amount. On increasing it, however, by rapid steps to thirty, forty, sixty, or even ninety grains three times a day, the case has responded immediately and *pari passu* to the original quantity of the drug. There are two patients in the hospital at the present time who are striking instances of this effect. In each the dose was pushed gradually to ninety grains three times a day, with marked beneficial effect, and I may add that the patients themselves express an unhesitating opinion upon this point. There is nothing new of course in the employment of large doses. Forty years ago the late Dr. ELLIOTSON used to give as much as two drachms three times a day,* and with remarkably good results. But of late years, as a general rule, the dose has been so moderate, that to many practioners the employment of ten grains at a time is only gradually arrived at, and with some caution. No doubt in a very large number of cases a comparatively small dose is all that is required, and in practice, therefore, it is well to begin with a dose of ten grains, and increase it if necessary. I feel tolerably sure from repeated experiments that the iodide may be used, if occasion require it, as freely as the bromide of potassium, and that the opportunity of doing great good in syphilitic nervous affections is nearly as often missed by the employment of inadequate doses of the former drug as used notoriously to happen in respect to epilepsy from the exhibition of two small doses of the latter. Symptoms of iodism, very like those of severe nasal catarrh, will occasionally present themselves, but just as frequently, so far as I have seen, when the amount of salt employed is small as when it is considerable, and it is very rare indeed that the use of the drug has to be given up on this account. Occasionally, too, a kind of large pustular acne is a disagreeable consequence of its administration, but this is not so common as where the bromide has been given.

Whether iodide of potassium, or mercury, or both be employed, a very long continuance of the treatment seems always to be required, and an immediate resumption of it at the slightest indication of relapse. In certain cases, arsenic and iron may be used with advanatage in addition to these means, bromide of potassium may be added in cases of convulsion, and electricity may be employed to help the nutrition of wasted muscles; but practically the treatment of this class of nervous affections is the treatment of the constitutional disorder."

*The Lancet, 1832.

